# A striking new species of leaf warbler from the Lesser Sundas as uncovered through morphology and genomics

**DOI:** 10.1038/s41598-018-34101-7

**Published:** 2018-10-23

**Authors:** Nathaniel. S. R. Ng, Dewi. M. Prawiradilaga, Elize. Y. X. Ng, Suparno, Hidayat Ashari, Colin Trainor, Philippe Verbelen, Frank. E. Rheindt

**Affiliations:** 1 Department of Biological Sciences, National University of Singapore, 14 Science Drive 4, Singapore, 129801 Singapore; 2 Division of Zoology, Research Center for Biology, Indonesian Institute of Sciences (LIPI), Jalan Raya Jakarta Bogor KM 46, Cibinong Science Center, Cibinong, 16911 Indonesia; 3 Research Institute for the Environment and Livelihoods, Charles Darwin University, Darwin, NT 0909 Australia; 4 Krijgsgasthuisstraat 89, 9000 Ghent, Belgium

## Abstract

Leaf warblers (Aves; Phylloscopidae) are a diverse clade of insectivorous, canopy-dwelling songbirds widespread across the Old World. The taxonomy of Australasian leaf warblers is particularly complex, with multiple species-level divergences between island taxa in the region requiring further scrutiny. We use a combination of morphology, bioacoustics, and analysis of thousands of genome-wide markers to investigate and describe a new species of *Phylloscopus* leaf warbler from the island of Rote in the Lesser Sundas, Indonesia. We show that this new Rote Leaf Warbler is morphologically and genomically highly distinct from its congenerics, but do not find vocal differentiation between different island taxa. We discuss the behaviour and ecology of this highly distinctive new species, and make recommendations about its conservation status. We believe this constitutes the first description of a novel bird species that is partly based on insights from massive amounts of genome-wide DNA markers.

## Introduction

Leaf warblers (Aves; Phylloscopidae) are an Old World clade of insectivorous, canopy-dwelling songbirds widespread across Europe and Australasia, with some species occurring in Africa^1^. These active, constantly-moving birds are found in forest and woodland, where they glean insects from the foliage^1^. Among songbirds, leaf warblers have undergone some of the most pronounced taxonomic upheaval in the past few decades^2–4^. The taxonomy of the Australasian clade of leaf warblers is particularly complex and poorly understood, and in need of detailed review^1,5^, with multiple species-level divergences between island taxa requiring further scrutiny^6–8^. A species-level phylogeny of the family was just published in 2018^9^.

**Figure 1:**
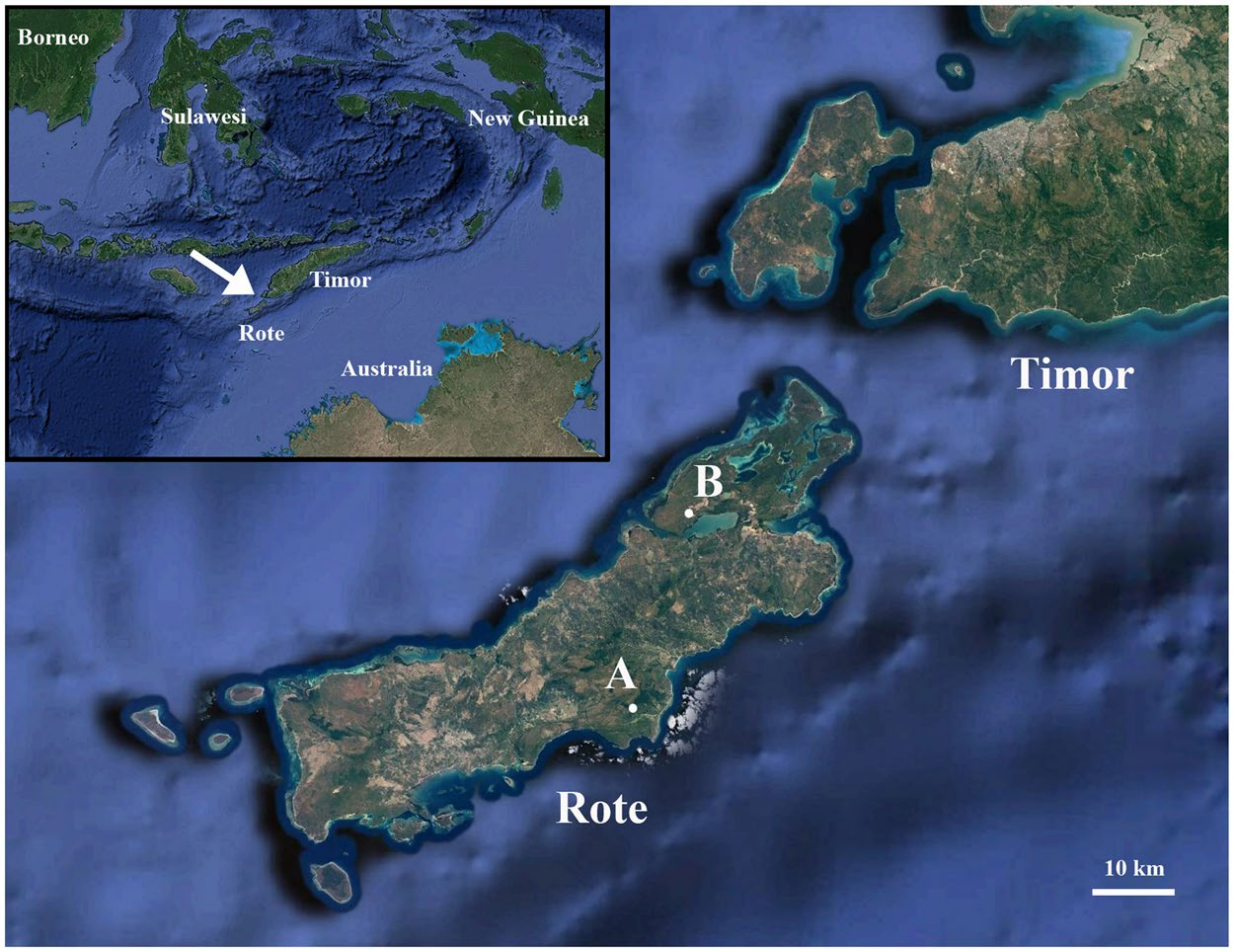
Maps showing location of Rote island in Wallacea (inset) and field localities on Rote Island: Seda forest (**A**) and woodland close to Bolatena village on the Tapuafu peninsula (**B**). Map data: Google, DigitalGlobe.

Most islands in the eastern Indonesian region of Wallacea are known to harbour breeding populations of leaf warblers, which are usually restricted to montane forests across the archipelago. Only on the more arid island of Timor and some of its satellites are leaf warblers known to descend to drier and lower elevations^4^. One of Timor’s largest satellites, the island of Rote (Fig. 1), is arid and low-lying, rising to a maximum of 420 m only. The presence of deep sea (>120 m below modern day sea level) between Rote and Timor ensures that these islands have never before been connected by Pleistocene land-bridges^10,11^, and Rote was historically not known to be inhabited by any leaf warbler population^12^. In December 2004, one of us (CT) was the first to report the presence of a leaf warbler from Rote when observing several birds on the Tapuafu peninsula in the northeast of the island (Fig. 1). The birds were “…frequent in woodlands and tropical dry forest…” throughout the peninsula, and uttered a “…breezy, rising and falling whistle…” not unlike that of leaf warblers on adjacent Timor, *P*. *presbytes*^13^.

Later, in 2009, PV visited Rote to observe and photograph the birds, and noticed for the first time that Rote leaf-warblers looked substantially different from any other Asian, African or European species he was familiar with. Among other morphological and colour differences, Rote leaf-warblers possessed a considerably longer bill than is typical for the entire family, with a yellow orange lower mandible, a broader and more yellow supercilium than in *P*. *presbytes*, a more prominent yellow crown stripe, warmer yellow sides of the head and underparts, and an olive-green rather than olive-grey crown^14^.

As part of an expedition to study this new taxon, some of us (DMP’s team, NSRN, FER) visited two sites on Rote between 29th November and 2nd December 2015. The first field site (10°47′14.32″S, 123°12′04.71″E; 414 m above sea level) was within Seda forest, which is one of the few small mature forest patches still remaining on Rote Island. The second field site was close to Bolatena village in the Tapuafu peninsula in northeastern Rote (10°35′26.21″S, 123°15′31.91″E; 22 m above sea level) (Fig. 1). The predominant vegetation cover at the latter site is disturbed forest. While the leaf warblers can be elusive when foraging during the day, the use of playback made it possible for us to observe them at both locations, where we paid close attention to their morphology, movements and behaviour. By using mist-nets, we collected a holotype specimen at the second field site on the Tapuafu peninsula in the evening of 30th November 2015. We employ a combination of morphological and biometric comparisons, bioacoustic analyses, and phylogenetic methods (using both mitochondrial and genome-wide markers) to describe this new species of *Phylloscopus* leaf warbler from Rote.

## Materials and methods

### Fieldwork

Collection was performed using mist-nets, which were set up at specific locations to target the leaf warbler. We do not believe that our collection of a single individual leaf warbler had a negative impact on the survival of this taxon. Fieldwork was conducted according to the government guidelines of the Republic of Indonesia and the Republic of Singapore. Nets were checked every half-hour, and closed in the evenings to minimize accidental trapping of non-target organisms. We obtained approval for our research practices from the Institutional Animal Care and Use Committee of the National University of Singapore. All specimens were obtained in the presence of personnel from Indonesia’s government agency LIPI. All mist-netting and collections were carried out with the requisite permits (see Acknowledgements).

### Morphological and Biometric Comparisons

Our baseline taxonomic treatment follows Eaton *et al*.^4^, with the exception that we retain Indonesian and Melanesian leaf warblers under the genus *Phylloscopus* Boie 1826, in accordance with recommendations by del Hoyo & Collar^1^ and Alström *et al*.^9^. The specimen of the new species that forms the basis of our examination is listed below as the holotype. Thirty-four specimens of closely-related, congeneric Sundaic, Wallacean, and Melanesian leaf warblers were examined at the Museum Zoologicum Bogoriense (MZB) in Bogor, Indonesia (Table 1). We took a range of measurements (bill to skull length, tarsus length, wing length, tail length, total length, wing spread, weight) of the Rote holotype as well as the two available specimens of the Timor Leaf Warbler *P*. *presbytes*, with which leaf warblers from Rote have been mooted to be conspecific based on geographical proximity. Tarsus length measurements were taken from the inner bend of the tibiotarsal articulation to the base of the toes, wing length measurements were taken with the wings stretched and flattened, and for total length measurements were taken after first laying the specimens on their backs and gently pressing the head and neck down. Bare parts colouration was noted in the field. Plumage colouration was noted using the Munsell colour notation system^15^.

**Table 1:** *Phylloscopus* specimens examined for morphological comparisons.

Name	Specimen number	Locality	Sex
Mountain Leaf Warbler (*P*. *trivirgatus trivirgatus*)	33.961	Gunung Ijen, East Java	?
	32.100	Central Java	M, juv
	5619	Central Java	M
	1758	Gunung Kerinci, Sumatra	M
	74.283	Gunung Gede, West Java	?
	8196	Lombok	M
	9809	Aceh, Sumatra	M
	33.950	Gunung Ijen, East Java	F
	32.099	Central Java	M
	32.182	Gunung Slamet, Central Java	M
North Moluccan Leaf Warbler (*P*. *waterstradti*)	26.330	Bacan	M
	34.080	Obi	F
Buru Leaf Warbler (*P*. *everetti*)	8194	Buru	M
	8195	Buru	M
	32.462	Buru	M
Seram Leaf Warbler (*P*. *ceramensis*)	33.255	Seram	M
	33.227	Seram	F
	33.251	Seram	F
Island Leaf Warbler (*P*. *poliocephalus poliocephalus*)	8202	Arfak mountains, West Papua	?
	8203	Arfak mountains, West Papua	M
Island Leaf Warbler (*P*. *p*. *giulianettii*)	25.824	Idenburg mountains, West Papua	M
	25.825	Lake Habema, West Papua	M
Timor Leaf Warbler (*P*. *presbytes*)	34.674	Gunung Mutis, West Timor	M
	34.673	Gunung Mutis, West Timor	F
Flores Leaf Warbler (*P*. *floresianus*)	775	West Flores	M
Sulawesi Leaf Warbler (*P*. *nesophilus*)	33.381	West Sulawesi	F
	32.901	Central Sulawesi	M
	33.538	Central Sulawesi	M
	32.902	Southeast Sulawesi	F
	33.426	West Sulawesi	M
	33.394	West Sulawesi	M
	33.427	West Sulawesi	F
Lompobattang Leaf Warbler (*P*. *sarasinorum*)	31.478	South Sulawesi	M
Rote Leaf Warbler (*P*. species nova)	34.652	Tapuafu peninsula, Pulau Rote	M

All specimens are from the Museum Zoologicum Bogoriense (MZB, Cibinong, West Java).

We used bill length as a proportion of total body length when comparing bill lengths between leaf warblers from Rote and nearby Timor, as doing so would allow us to confirm that any observed differences in bill length are not merely due to body size differences.

### Genomic Sampling Regime and DNA Extraction

DNA was extracted from the muscle samples of four specimens we collected ourselves as well as additional muscle samples from the American Museum of Natural History (AMNH) in New York (Table 2). Samples we collected ourselves comprise the holotype specimen from Rote (henceforth referred to as the Rote Leaf Warbler), two individuals of Timor Leaf Warbler *P*. *presbytes* (West Timor), and one individual of a leaf-warbler population from Peleng provisionally assigned to the Sulawesi Leaf Warbler *P*. *nesophilus* (henceforth referred to as the Peleng Leaf Warbler)^4^ collected on an earlier expedition in 2013^16^. Tissue vouchers for all self-collected specimens are deposited at the MZB (Table 2). Muscle samples from the AMNH comprise two individuals of the Kolombangara Leaf Warbler *P*. *amoenus* from Kolombangara in the Solomon Islands, three individuals of Lompobattang Leaf Warbler *P*. *sarasinorum* from South Sulawesi, one individual of Island Leaf Warbler *P*. *poliocephalus giulianettii* from Karkar island in Papua New Guinea (henceforth the Island Leaf Warbler ssp. *giulianettii*), and two individuals of Island Leaf Warbler *P*. *poliocephalus pallescens* from Kolombangara in the Solomon Islands (henceforth the Island Leaf Warbler ssp. *pallescens*). Extraction was performed using the DNEasy extraction kit from Qiagen (Venlo, Netherlands) following manufacturer’s instructions. We eluted the DNA in two consecutive rounds of 100 μl of molecular-grade water.

**Table 2:** Taxa sampled for genomic analysis, localities, and genomic read information.

Taxon	Specimen voucher number (Locality)/Sample name	Total reads	Retained reads	Percentage Retained reads
Rote Leaf Warbler (*P*. species nova)	MZB.Ornit.34.652 (Rote Island)/NTT107	2,591,470	2,541,128	98%
Timor Leaf Warbler (*P*. *presbytes*)	MZB. Ornit.34.673 (West Timor)/NTT139	3,821,262	3,759,719	98%
	MZB. Ornit.34.674 (West Timor)/NTT140	3,439,488	3,372,939	98%
Peleng Leaf Warbler (*P*. *“nesophilus”* tax. novum)	MZB. Ornit.34.440 (Peleng Island)/PEL14	2,640,380	2,275,646	86%
Lompobattang Leaf Warbler (*P*. *sarasinorum*)	AMNH DOT12560 (Gowa Regency, South Sulawesi)/24628PS	2,539,636	2,174,904	86%
	AMNH DOT12559 (Gowa Regency, South Sulawesi)/24629PS	3,472,528	3,127,453	90%
	AMNH DOT12560 (Mt Lompobattang, South Sulawesi)/24634PS	3,543,948	3,083,575	87%
Kolombangara Leaf Warbler (*P*. *amoenus*)	AMNH DOT279 (Mt Mbatuvana, Kolombangara Is.)/279PA	3,022,962	2,677,349	89%
	AMNH DOT251 (Mt Mbatuvana, Kolombangara Is.)/251PA	4,119,908	3,719,027	90%
Island Leaf Warbler (*P*. *poliocephlaus pallescens*)	AMNH DOT220 (Mt Mbatuvana, Kolombangara Is.)/220PP	3,332,630	2,878,066	86%
	AMNH DOT266 (Mt Mbatuvana, Kolombangara Is.)/266PP	3,735,222	3,338,001	89%
Island Leaf Warbler (*P*. *poliocephalus giulianettii*)	AMNH DOT19835 (Crater Rim Bush camp, Karkar Is.)/19835PT	3,751,110	2,638,266	70%

Abbreviations: MZB (Museum Zoologicum Bogoriense, Cibinong, West Java), AMNH (American Museum of Natural History, New York).

### Genomic Library Preparation and Sequencing

We prepared double-digest restriction-site associated DNA sequencing (ddRADSeq) libraries using a modified version of Peterson *et al*.‘s^17^ protocol, as described by Garg *et al*.^18^ Libraries were prepared for the four samples we collected ourselves as well as all the samples from AMNH. We used the restriction enzymes EcoRI and MspI, and performed DNA fragment cleanup steps with Sera-Mag magnetic beads (Thermo-Scientific, Fremont, California). Final concentration and fragment size checks on the library were performed using a Qubit 2.0 Broad Range DNA assay (Invitrogen, California, USA) and a Fragment Analyser (Advanced Analytical Technologies, Iowa, USA), respectively. Sequencing was performed on a 150 bp paired-end Illumina HiSeq2500 run at the Singapore Center for Environmental Life Sciences Engineering.

### Genomic Data Filtering and Processing

We used STACKS 1.34^19^ to demultiplex the genomic data. We removed all reads with one or more low quality (Phred score <10) or uncalled base(s), and truncated all reads to 145 bp. We allowed for up to one mismatch across barcodes.

We used the genome of the closely related Greenish Warbler *P*. *trochiloides viridanus* (mapped to the complete zebra finch *Taeniopygia guttata* genome) as our reference genome^20^. We indexed the reference genome and aligned our sequence reads using the Burrows-Wheeler Aligner 0.7.12^21^ and samtools 0.1.19 (http://samtools.sourceforge.net), removing all reads with MAPQ scores lower than 20. We then generated three different genomic datasets for downstream analyses. The first is a three-taxon SNP dataset containing the Rote Leaf Warbler, the Timor Leaf Warbler, and the Peleng Leaf Warbler (the latter functioning as an outgroup). This first SNP dataset focuses on the Rote – Timor Leaf Warbler taxon pair, allowing us to better assess levels of divergence between these two geographically adjacent taxa on the basis of a higher proportion of diagnostic SNPs. The second and third datasets comprise all seven taxa we sequenced: one a SNP dataset, and the other a concatenated read supermatrix. These second and third datasets provide more resolution into the deeper, more ancestral phylogenetic relationships among Southeast Asian *Phylloscopus* warblers.

We performed SNP calling for the three-taxon and seven-taxon SNP datasets using the ref_map and populations modules from STACKS 1.34. We set the minimum required stack depth per read at five, allowed for up to two mismatches between stacks within each individual, and allowed for up to two mismatches between stacks across individuals. All loci had to be present in all individuals from all populations. We retained a single random SNP from each locus. We then used the program PLINK 1.9^22^ to filter SNPs for strongly linked loci, the inclusion of which may affect downstream analyses^23–26^. For this filtering, we used a sliding window of 25 SNPs, a step size of 10, and an r^2^ threshold of 0.9. We then used BayeScan 2.1^27^ to filter the SNP sets for loci under strong selection at a 5% false discovery rate.

We generated the seven-taxon concatenated read supermatrix using the program pyRAD^28^ on the basis of the demultiplexed reads from STACKS. We set the minimum required stack depth to five, discarded any read with more than four bases having Phred scores below 20, and kept the within and between read clustering thresholds at 0.88.

### Population Divergence Analysis and Phylogenomic Inference

In order to infer population genomic divergence between the Rote and Timor Leaf Warblers, we performed Principal Component Analysis (PCA) on the three taxon SNP dataset. We performed this analysis in R version 3.3.1^29^ using the ade4^30^, factoextra^31^ and ggplot2^32^ packages.

We employed two different methods to infer phylogenetic relationships between the Rote Leaf Warbler and other Southeast Asian leaf warblers: mitochondrial gene analysis as well as Maximum Likelihood (ML) tree inference on the concatenated genomic read supermatrix.

For mitochondrial gene analysis, we amplified and sequenced the cytochrome*-b* (cytb) gene of four samples (the Rote Leaf Warbler, the two Timor Leaf Warblers, and the Peleng Leaf Warbler), then compared them with cytb sequences of additional taxa and a greenish warbler *P*. *trochiloides* outgroup^6^ downloaded from GenBank (Table 3). Mitochondrial gene amplification was performed using the primers H16065^33^ and L14995^34^, with PCR carried out for 40 cycles, using an annealing temperature of 72 °C. We used ExoSAP-IT (Thermo-Fisher, Massachusetts, USA) for PCR product cleanup, and cycle-sequencing was performed using the BigDye Terminator v3.1 Cycle sequencing kit (Applied Biosystems Inc., California, USA). We assembled DNA sequences with CodonCode Aligner version 4.1 (www.codoncode.com). We carried out sequence alignment in MEGA7.0.2^35^ using the ClustalW algorithm^36^. The final cytb alignment was 1,100 bp in length.

**Table 3:** Samples and sequences used in the cytochrome-b mitochondrial gene analysis.

Taxon	Locality	GenBank Accession #	Reference
Rote Leaf Warbler (*P*. species nova)	Rote Island	—	Present study
Timor Leaf Warbler (*P*. *presbytes*)	West Timor	—	Present study
	West Timor	—	Present study
Peleng Leaf Warbler (*P*. *“nesophilus”* tax. nov.)	Peleng Island	—	Present study
Lompobattang Leaf Warbler (*P*. *sarasinorum*)	South Sulawesi, Indonesia	AY656240	^6^
Mountain Leaf Warbler (*P*. *trivirgatus trivirgatus*)	Luzon, Philippines	L77145	^51^
	Java, Indonesia	AY656244	^6^
Island Leaf Warbler (*P*. *poliocephalus giulianettii*)	Papua New Guinea	AY656224	^6^
Kolombangara Leaf Warbler (*P*. *amoenus*)	Kolombangara, Solomon Islands	AY887676	^6^
Greenish Warbler (*P*. *trochiloides viridanus*)	Tianjin, China	HQ608824	^52^

We used the mitochondrial gene data to construct phylogenetic trees using both ML and maximum parsimony (MP) methods. Both mitochondrial tree inference analyses were run on MEGA7.0.2 using partial deletion of gaps. MP was run with standard bootstrap of 10,000 replicates and with the tree bisection and reconnection method^37^. Sectorial, ratchet and tree-fusing parameters were set to default. For ML analysis, we first used jModelTest^38^ to determine the best performing evolutionary model, which was determined to be the Jukes-Cantor model^39^. MEGA7.0.2 was then used to perform ML analysis. We calculated absolute pairwise distances and estimated standard error using 10,000 bootstrap replicates.

For the genomic tree analysis based on the read supermatrix, we employed RAxML v8.2.9^40^ to perform ML tree inference using the invariant site + gamma + General-Time-Reversible model (GTRGAMMA)^41^ and 1000 rapid bootstrap searches. Final tree results were visualized using FigTree v 1.4.2 (available at http://tree.bio.ed.ac.uk/software/figtree/).

### Bioacoustic Analyses

A total of 176 *Phylloscopus* leaf warbler recordings from the Lesser Sundas (Atauro, Flores, Rote, Timor, Wetar) were compiled and – after removal of low-quality recordings – measurements were conducted for 153 recordings, totalling 33 calls and 135 songs. Despite our best efforts, we were unable to obtain a recording of the holotype individual. Songs and calls constitute different, non-homologous vocalizations and were therefore measured and analysed separately. Songs can be described as complicated jumbles of melodious notes and typically consist of multiple elements, whereas calls are chippy and relatively simple in comparison, although always clearly multi-syllabic. For each recording, vocalizations were classified and categorized as calls or songs. Ninety six recordings were made in the field by ourselves and an additional 80 recordings were obtained from online repositories (Supplementary File 1): AVoCet (http://avocet.zoology.msu.edu/), Internet Bird Collection (http://www.hbw.com/ibc), and Xeno-Canto (http://www.xeno-canto.org/), and were all converted to WAV format for analysis.

Sonograms were visualised and analysed with Raven Pro v1.5 (Bioacoustics Research Program, Cornell Laboratory of Ornithology, Ithaca, NY, USA). Contrast and brightness were set to equal levels across recordings and sharpness was set to 500; all other settings were left at default.

**Figure 2:**
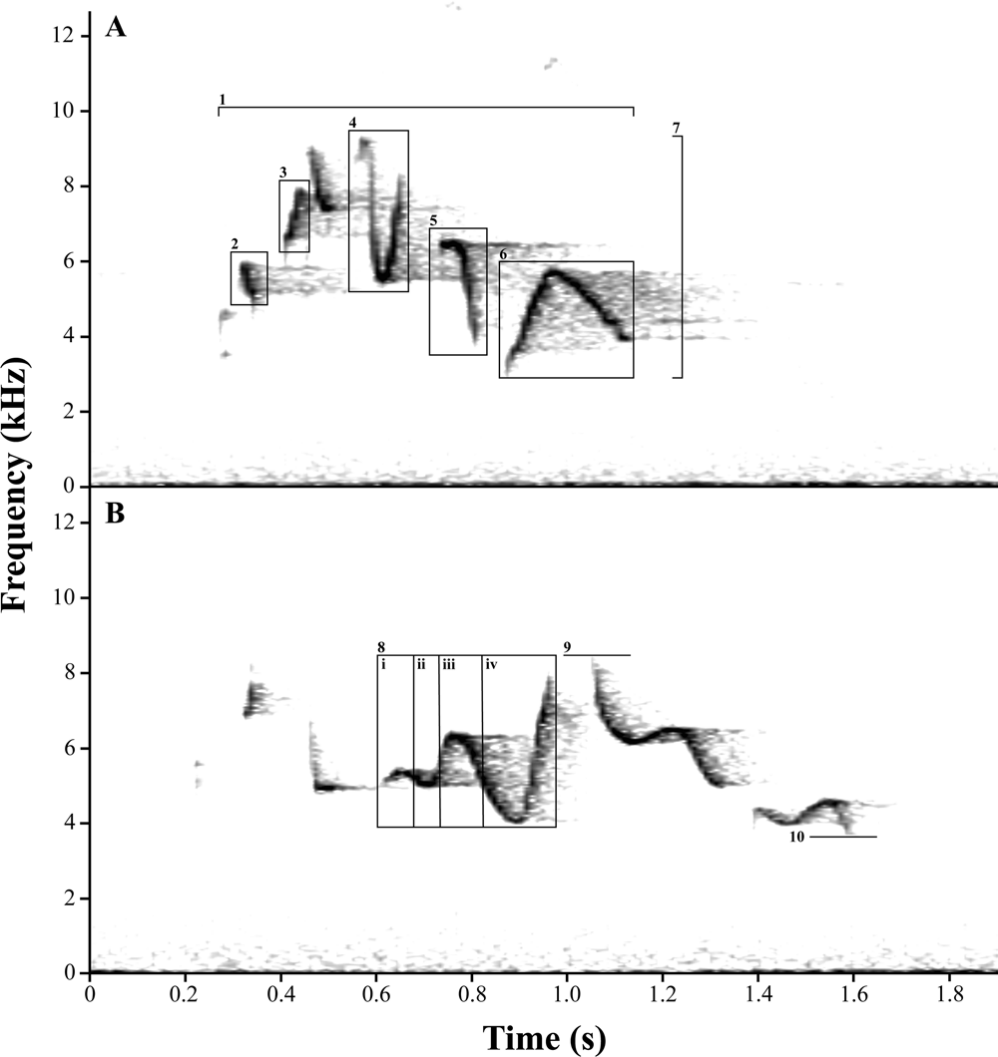
Sonogram of two song types (**A**–**B**) from recording XC302571 (Timor), with each panel depicting a single strophe. Vocal characters: (1) strophe duration, (2) an example of a descending element, (3) an example of an ascending element, (4) an example of a V-shaped element, (5) another example of a descending element, (6) an example of a ∧-shaped element, (7) bandwidth frequency of a strophe, (8) an example of a complex element that is further divided into four parts, (i and iii) ∧-shaped, (ii and iv) V-shaped, (9) highest frequency of a strophe, (10) lowest frequency of a strophe.

We defined an element as an unbroken segment of a vocalization within a strophe (Fig. 2A). All populations were characterized by great variation in songs, even within individuals, that ranged between three to twelve elements per strophe (Fig. 2). Calls were less variable, with element counts between three to seven per strophe. A total of 19 parameters were analysed (Fig. 2): (1) number of elements per strophe; (2) strophe duration; (3) lowest frequency of a strophe; (4) highest frequency of a strophe; (5) bandwidth frequency of a strophe; (6) proportion of ascending elements in a strophe; (7) proportion of descending elements in a strophe; (8) proportion of V-shaped element parts in a strophe; (9) proportion of ∧-shaped element parts in a strophe; (10) lowest frequency of the longest element in a strophe; (11) highest frequency of the longest element in a strophe; (12) bandwidth frequency of the longest element in a strophe; (13) duration of the longest element in a strophe; (14) duration of longest element in proportion to the entire strophe; (15) lowest frequency of the final element in a strophe; (16) highest frequency of the final element in a strophe; (17) bandwidth frequency of the final element in a strophe; (18) duration of the final element in a strophe; and (19) duration of final element in proportion to the entire strophe. Songs were measured for the first nine parameters whereas all 19 parameters were measured for calls. In all, 1,095 strophes (981 from songs, 114 from calls) and 7,446 constituent elements (6,847 from songs, 599 from calls) were analysed.

We took measurements of multiple song and call motifs for each individual. Means of each individual were then calculated for the respective vocalization type (call and/or song). In the end, our extensive collection of recordings allowed us to base individual means on an average of 7.3 song strophes and 3.5 call strophes per individual. PCA was conducted, using the calculated means, with the prcomp function in R, version 3.3.1^24^. We have included examples of sound files of both the song and call of the Rote Leaf Warbler in the Supplementary Material (Supplementary Files 2 and 3 respectively).

## Results

On the basis of our morphological, biometric, and genomic analyses, we here describe the unnamed population of leaf warbler from Rote Island as a new species:

*Phylloscopus rotiensis*, species nova

English name: Rote Leaf Warbler

ZooBank registration ID: BF79FD8C-E863-44B7-A901-3744A2DF82D5 Holotype: Museum Zoologicum Bogoriense, MZB.Ornit.34.652. Adult male, no body or wing moult, no brood patch (feathering over), all feathers fresh; collected on 30th November 2015 by FER, prepared as a dry skin by Suparno; tissue samples of pectoral muscle and liver taken and deposited at the MZB. Type locality: dry woodland close to Bolatena village in *kecamatan* (=district) Rote-Timur, *kabupaten* (=regency) of Rote Ndao, on Rote Island, in Nusa Tenggara Timur Province, Indonesia. Coordinates: 10°35′26.21″S, 123°15′31.91″E

Etymology: We name this species after Rote Island, the only locality at which this leaf warbler can be found.

Holotype description: Measurements (from the field): weight 7.5 g, wing length 57 mm, wing spread 155 mm, total length 103 mm, bill length 16.3 mm, tail length 38 mm, tarsus length 20.2 mm.

Bare parts coloration: Lower mandible yellow-orange. Culmen horn, becoming blackish horn toward base and tip. Iris dark brown. Tarsus horn with yellow suffusion.

Plumage coloration: Crown: dark olive-grey (Munsell hue 10Y 2/4). Median crown stripe: pale lemon yellow (Munsell 10Y 9/6); starts at base of culmen, extending to the nape. Lores: dark olive grey (Munsell 5Y 2/2), forming an eye stripe from base of bill through the eye along the sides of the head above the ear coverts. Ear coverts: pale lemon yellow (Munsell 10Y 9/6), with slight dark olive grey grizzling (Munsell 5Y 2/2). Supercilium, chin, throat, breast, belly, flanks: lemon yellow, but slightly more saturated than on median crown stripe (Munsell 7.5Y 9/8); brighter lemon yellow on mid-breast and mid-belly (reaching Munsell 7.5Y 10/8). Nape, mantle, scapulars, rump, upper tail coverts olive-grey (Munsell 10Y 4/6), slightly paler than on the rectrices and remiges. Retrices and remiges darker olive-grey (Munsell 10Y 3/4) than nape, mantle, scapulars, rump, and uppertail coverts. Outermost tail feathers on each side (T5 and T6) have white inner vanes and dark olive grey outer vanes. Remiges: pale cream margins on greater coverts forming a single narrow wingbar (Munsell 10Y 9/2). White underwing coverts, grading to cream (Munsell 10Y 3/4) and then pale lemon yellow (Munsell 7.5Y 10/8) along the leading edge of the underwing between the bend of the wing and the alula. Dark olive grey underside to primaries (Munsell 10Y 4/4) with whitish inner edge.

### Diagnosis

The new taxon is distinguished from all other *Phylloscopus* leaf warblers by a proportionately much longer bill.

**Figure 3:**
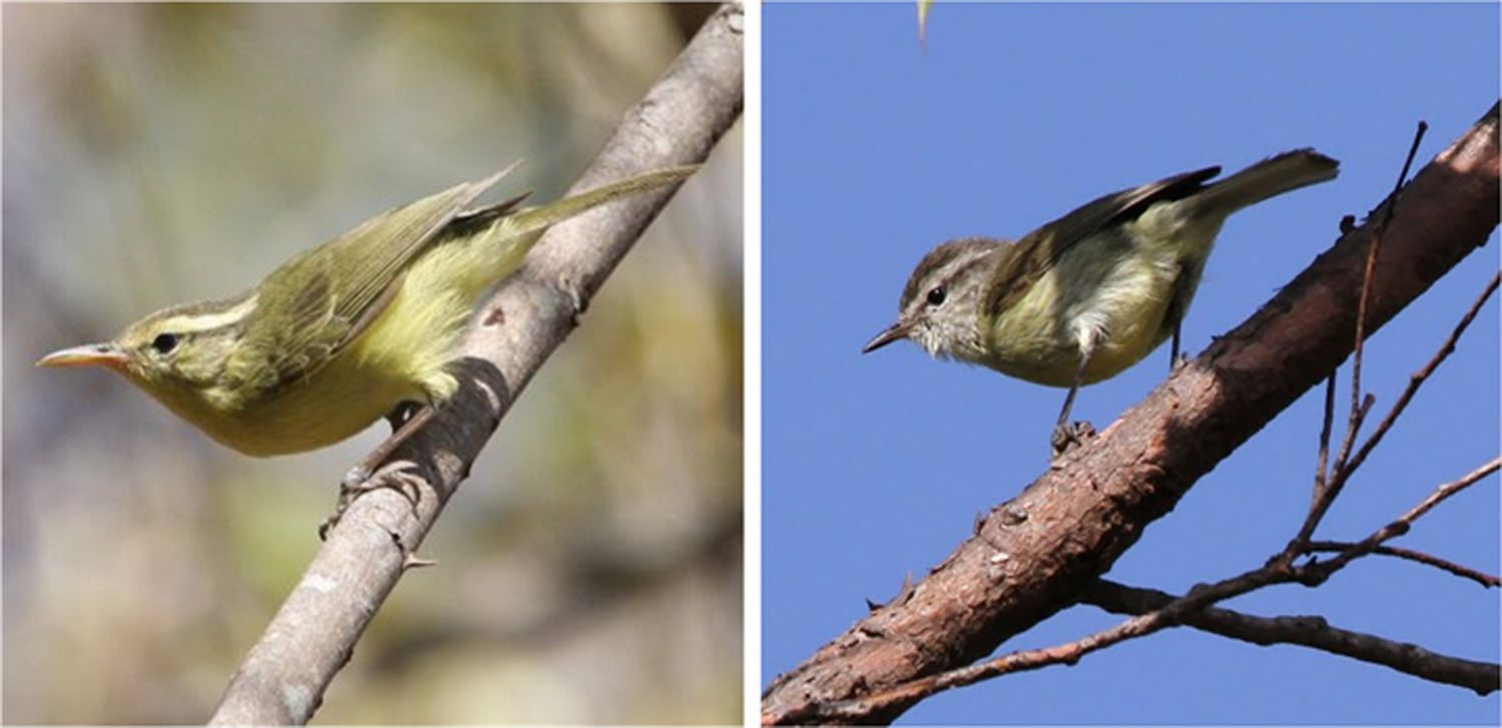
Pictures from the field of the newly described Rote Leaf Warbler (on the left; PV) and the Timor Leaf Warbler *P*. *presbytes* from Mount Ramelau in Timor Leste (on the right; CT). The unusually long, colourful bill and overall more saturated yellowish plumage of the Rote Leaf Warbler are apparent.

The Rote Leaf Warbler specimen had a bill length of 16.3 mm, as compared to 13.4 ± 0.1 mm in the Timor Leaf Warbler (*n* = 2). A comparison of bill length as a proportion of wing length between the Rote and Timor Leaf Warblers indicates that the Rote Leaf Warbler shows a bill to wing length ratio of 0.286, whereas the Timor Leaf Warbler has a bill to wing length ratio of merely 0.248 ± 0.01. The bill of the Rote Leaf Warbler is thus proportionately 15% longer than that of the Timor Leaf Warbler (Fig. 3).

#### Versus Mountain Leaf Warbler (*P*. *trivirgatus*, specimens from west of Wallace’s Line)

The Rote Leaf Warbler can be distinguished from the Mountain Leaf Warbler by its overall less saturated plumage colour (duller olive on back, duller yellow on body, and a duller yellow supercilium and median crown stripe). The Roti Leaf Warbler also has a yellow-orange lower mandible, as compared to the Mountain Leaf Warbler’s mostly dark lower mandible. Also distinguished by the Mountain Leaf Warbler’s lack of a wingbar and lack of white on the tail feathers (T5 and T6).

#### Versus Timor Leaf Warbler *P*. *presbytes*, Timor

The Rote Leaf Warbler has a much stronger median crown stripe, which is absent or very faint in the Timor Leaf Warbler. The Timor Leaf Warbler also exhibits an overall duller coloration, with paler yellow underparts, supercilium and ear coverts, and duller olive-brown upperparts. The Rote Leaf Warbler also has a wholly yellow-orange lower mandible, as compared to the mostly dark lower mandible of the Timor Leaf Warbler.

#### Versus Flores Leaf Warbler *P*. *floresianus*, Flores

The Rote Leaf Warbler has a much more prominent median crown stripe than the Flores Leaf Warbler, in which the crown stripe is absent or very faint. Overall plumage colour is more saturated in the Flores Leaf Warbler: the upperparts are of a richer olive-brown hue, and the underparts are a richer yellow (orange-yellow versus lemon yellow in the Rote Leaf Warbler). The Flores Leaf Warbler’s lower mandible is also dark-tipped, as compared to the Rote Leaf Warbler’s wholly yellow-orange lower mandible.

#### Versus Lompobattang Leaf Warbler *P*. *sarasinorum*, South Sulawesi

The underparts of the Rote Leaf Warbler are of a rich lemon-yellow, versus the much lighter pale yellow wash of the Lompobattang Leaf Warbler’s underparts. The upperparts of the Rote Leaf Warbler, in contrast, are paler as compared to the richer olive brown hue seen in the Lompobattang Leaf Warbler. Additionally, the Rote Leaf Warbler has a richer yellow supercilium and median crown stripe. The Lompobattang Leaf Warbler also has a mostly dark lower mandible, as compared to yellow-orange in the Rote Leaf Warbler.

#### Versus North Moluccan Leaf Warbler *P*. *waterstradti*, specimens from Bacan and Obi

The Rote Leaf Warbler’s median crown stripe distinguishes it from the North Moluccan Leaf Warbler, which has no such crown stripe. The Rote Leaf Warbler also has a yellow supercilium and throat (as compared to white in the North Moluccan Leaf Warbler), and tail feathers with white inner vanes (as compared to wholly olive brown in the North Moluccan Leaf Warbler). The Rote Leaf Warbler also has paler olive green upperparts, paler and less saturated yellow underparts, and a yellow orange lower mandible (versus the mostly dark lower mandible of the North Moluccan Leaf Warbler).

#### Versus Buru Leaf Warbler *P*. *everetti*, Buru

The Rote Leaf Warbler has a median crown stripe which is absent in the Buru Leaf Warbler. The two species also differ in the colour of the supercilium and throat: these are yellow in the Rote Leaf Warbler instead of white and pale grey respectively in the Buru Leaf Warbler. The underparts of the Rote Leaf Warbler are also of a paler yellow hue as compared to the bright sulphur yellow of the Buru Leaf Warbler. Finally, the Buru Leaf Warbler’s lower mandible is mostly dark.

#### Versus Seram Leaf Warbler *P*. *ceramensis*, Seram and Ambon

The Rote Leaf Warbler’s yellow median crown stripe differs from that of the Seram Leaf Warbler, which is olive. The Seram Leaf Warbler also has a whitish supercilium and throat (versus yellow in the Rote Leaf Warbler), and brighter yellow underparts. The outermost tail feathers also lack the white inner vanes which can be found in the Rote Leaf Warbler. Finally, the Rote Leaf Warbler’s yellow orange lower mandible differs from the mostly dark lower mandible of the Seram Leaf Warbler.

#### Versus Sulawesi Leaf Warbler *P*. *nesophilus*, specimens from West, Central, and Southeast Sulawesi

The Rote Leaf Warbler’s lemon-yellow underparts differ from the olive-yellow breast and vent of the Sulawesi Leaf Warbler. The Sulawesi Leaf Warbler also has darker brownish-olive upperparts, a faint or absent median crown stripe, a paler supercilium, a mostly dark lower mandible, and no white inner vanes on its outermost tail feathers.

#### Versus Island Leaf Warbler *P*. *poliocephalus giulianettii*, Papua New Guinea

The Rote Leaf Warbler has paler olive uperparts as compared to the Island Leaf Warbler ssp. *giulianettii*. The Rote Leaf Warbler also has a richer yellow supercilium, a yellow throat (as compared to the whitish chin and throat of the Island Leaf Warbler ssp. *giulianettii*), a yellow orange lower mandible, and less saturated yellow underparts.

#### Versus Island Leaf Warbler *P*. *poliocephalus poliocephalus*, Papua New Guinea

The Rote Leaf Warbler has a yellow supercilium, chin, throat, and ear coverts, which are all white in the Island Leaf Warbler sso. *poliocephalus*. The two can further be distinguished by the Rote Leaf Warbler’s median crown stripe (which is absent in the Island Leaf Warbler ssp. *poliocephalus*) and yellow orange lower mandible (mostly dark in the Island Leaf Warbler ssp. *poliocephlaus*). The Island Leaf Warbler ssp. *poliocephalus* also has brighter yellow underparts, darker olive upperparts, and lacks the white inner vanes on outermost tail feathers observed in the Rote Leaf Warbler.

### Genomic Data

We obtained more than 40 million 150-bp reads from across the 12 sequenced genomic samples (Table 3). After quality filtering, this number dropped to 35.5 million reads, corresponding to an overall retention rate of ~89%. For the smaller three-taxon genomic dataset, we obtained 12.5 million total reads out of which 11.9 million reads were retained (~96% retention rate).

After SNP calling, we obtained a total of 2,745 and 5,833 SNPs for the seven-taxon and three-taxon SNP datasets respectively, each with no missing data. The concatenated read dataset totalled 1,443,756 bp in length, assembled from 10,064 loci.

### Population Structure

**Figure 4:**
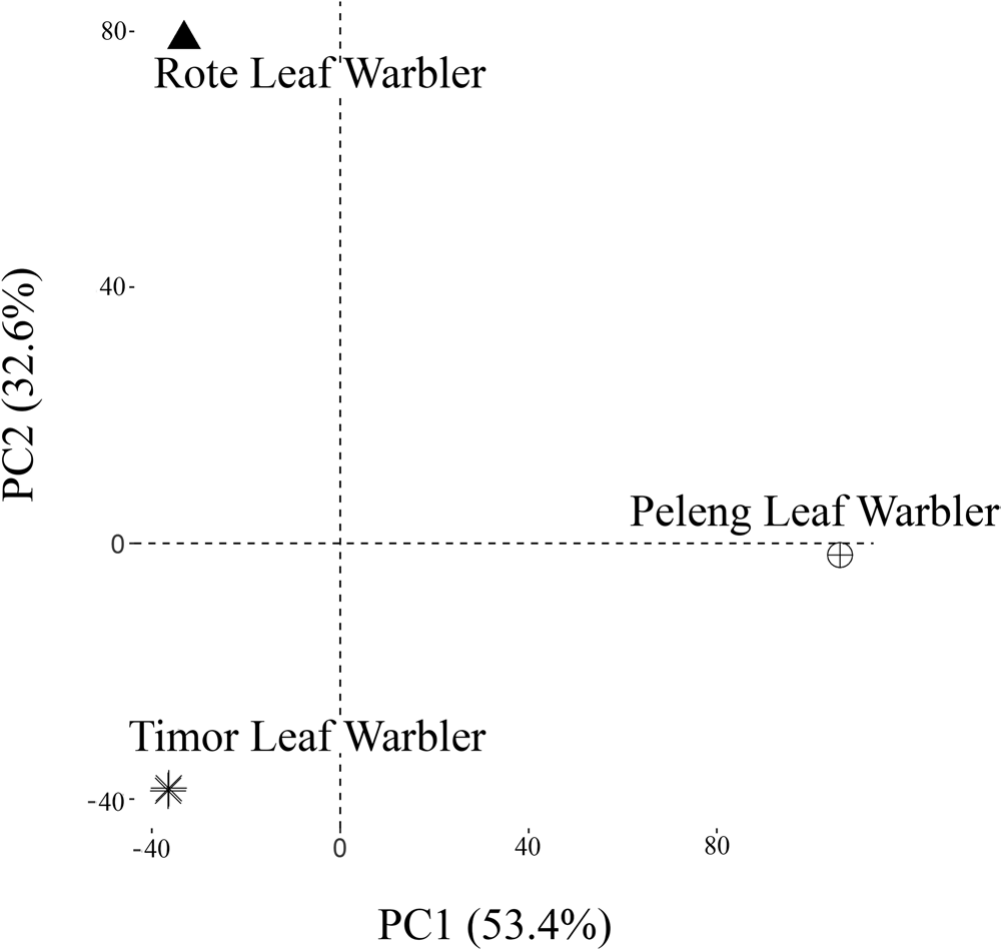
Results from PCA analysis show leaf warbler populations from Peleng (*n* = 1), Rote (*n* = 1) and Timor (*n* = 2) to be genomically distinct. PC1 and PC2 refer to Principal components 1 and 2 respectively.

PCA on the three taxon SNP dataset yielded two PCs which each accounted for more than 30% of observed variation. Together, PC1 and PC2 accounted for 86% of observed variation (Fig. 4). The Rote Leaf Warbler, Timor Leaf Warbler, and Peleng Leaf Warbler each formed separate, highly distinct clusters. PC1 (~53.4% of observed variation) clearly separates the Peleng Leaf Warbler from the two Nusa Tenggara taxa, while PC2 (~ 32.6% of observed variation) clearly separates the Rote and Timor Leaf Warblers from each other (Fig. 4).

### Phylogenetic Analysis

Uncorrected pairwise distances, distances calculated under the Jukes-Cantor model, and distances calculated under the General-Time-Reversible (GTR) models all yielded congruent results showing the Rote Leaf Warbler to be 3.6% mitochondrially divergent from the adjacent Timor Leaf Warbler (Table 4). Using the widely-followed mitochondrial clock rate of 2.1% per million years^42,43^, the Rote Leaf Warbler would be estimated to have diverged from the Timor Leaf Warbler roughly 1.7 million years ago. These divergence time estimates well correspond to those for the entire Indo-Malayan leaf warbler clade, which is estimated to have occurred about 2.0–2.5 million years ago^9^. It is about 5–6% divergent from all the other Wallacean taxa in our analysis.

**Table 4:** Pairwise uncorrected p-divergences on the basis of 1,162 bp long mitochondrial cytochrome-b sequences between the Rote Leaf Warbler, Timor Leaf Warbler, and other Indo-Papuan leaf warblers.

	Island Leaf Warbler ssp. *giulianettii*	Lompo-battang leaf warbler	Mountain Leaf Warbler (Java)	Kolom-bangara leaf warbler	Mountain Leaf Warbler (Luzon)	Rote Leaf Warbler	Timor Leaf Warbler	
Island Leaf Warbler ssp. *giulianettii*								
Lompobattang Leaf Warbler	4.7%							
Mountain Leaf Warbler (Java)	5.1%	4.6%						
Kolombangara Leaf Warbler	3.8%	4.3%	5.4%					
Mountain Leaf Warbler (Luzon)	5.4%	4.6%	5.4%	4.6%				
Rote Leaf Warbler	6.0%	5.5%	5.5%	5.2%	6.1%			
Timor Leaf Warbler	5.2%	5.0%	4.7%	5.1%	5.8%	**3.6%**		
	5.4%	5.2%	4.9%	5.3%	6.0%	**3.6%**	0.2%	
Peleng Leaf Warbler	5.0%	1.9%	4.9%	4.2%	4.4%	5.2%	5.1%	5.3%

The Rote and Timor Leaf Warblers are about 3.6% mitochondrially diverged from each other. For scientific names of taxa, see Table 3.

#### Both mitochondrial cytb tree inference analyses (ML and MP) returned the same topology, showing two highly supported (bootstrap >90) clades

**Figure 5:**
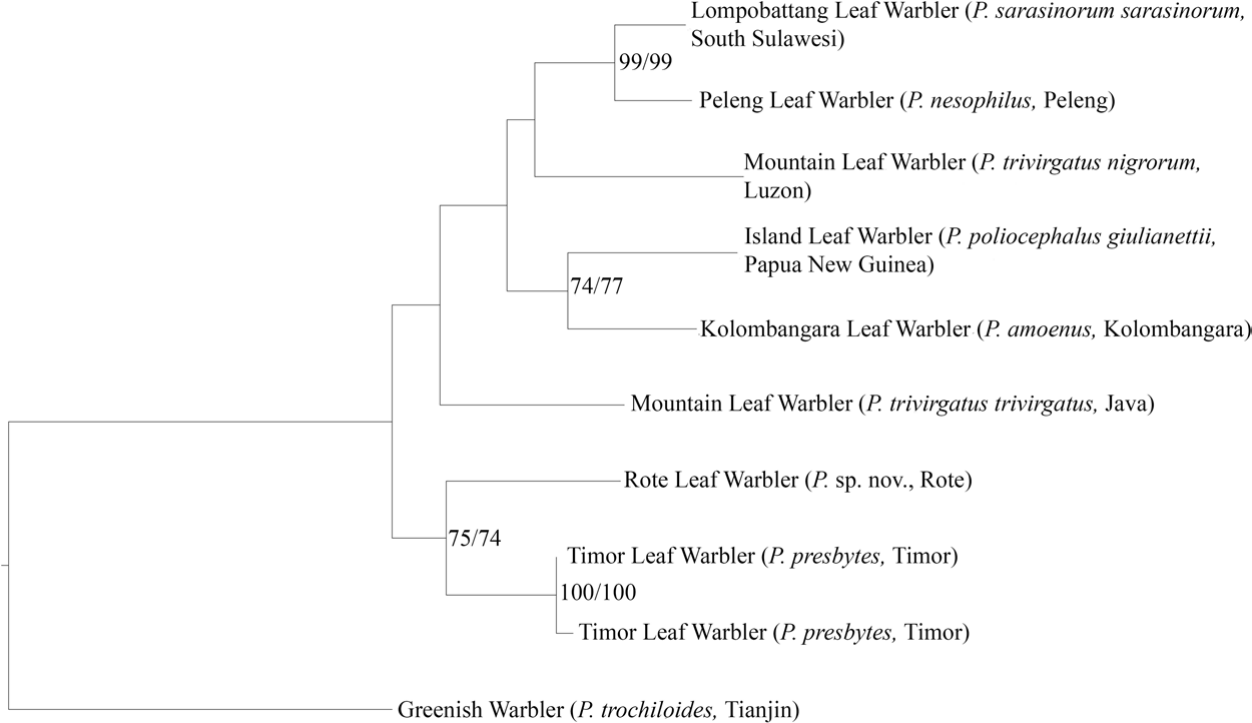
Results of maximum likelihood (ML) tree inference as applied to the mitochondrial cytochrome-b locus. Both ML and maximum parsimony (MP) returned identical tree topologies. The ML tree is used here, and branch lengths represent relative genetic distance. Node support higher than 70 is shown and represents values from ML and MP analyses respectively. The Timor Leaf Warblers cluster together with high branch support to the exclusion of the Rote Leaf Warbler.

one placing the two Timor Leaf Warblers together, and one placing the Peleng Leaf Warbler sister to the Lompobattang Leaf Warbler from South Sulawesi. All other nodes are poorly supported (Fig. 5).

**Figure 6:**
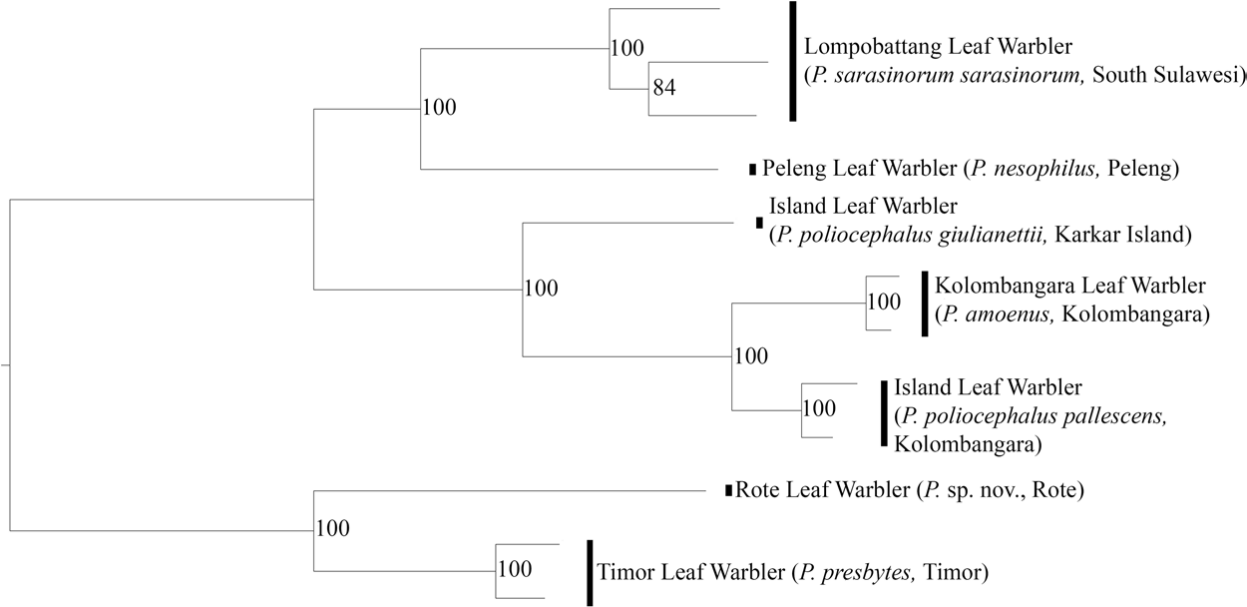
Maximum Likelihood tree from concatenated genomic read data (1,443,756 bp from 10,064 loci). Branch lengths reflect relative genetic distance. Branch support values higher than 70 are shown.

The maximum likelihood analysis performed on the concatenated read supermatrix (Fig. 6) resulted in a highly supported tree which shows concordance with the mitochondrial cytb tree (Fig. 5). The two Lesser Sunda populations (=Timor and Rote Leaf Warblers) emerge as sister to each other in a highly supported clade. We rooted the tree using the clade containing the Rote and Timor Leaf Warblers in accordance with evidence from the mitochondrial tree inference showing this clade to be sister to all the other Southeast Asian *Phylloscopus* in our analysis.

### Bioacoustic Analyses

**Figure 7:**
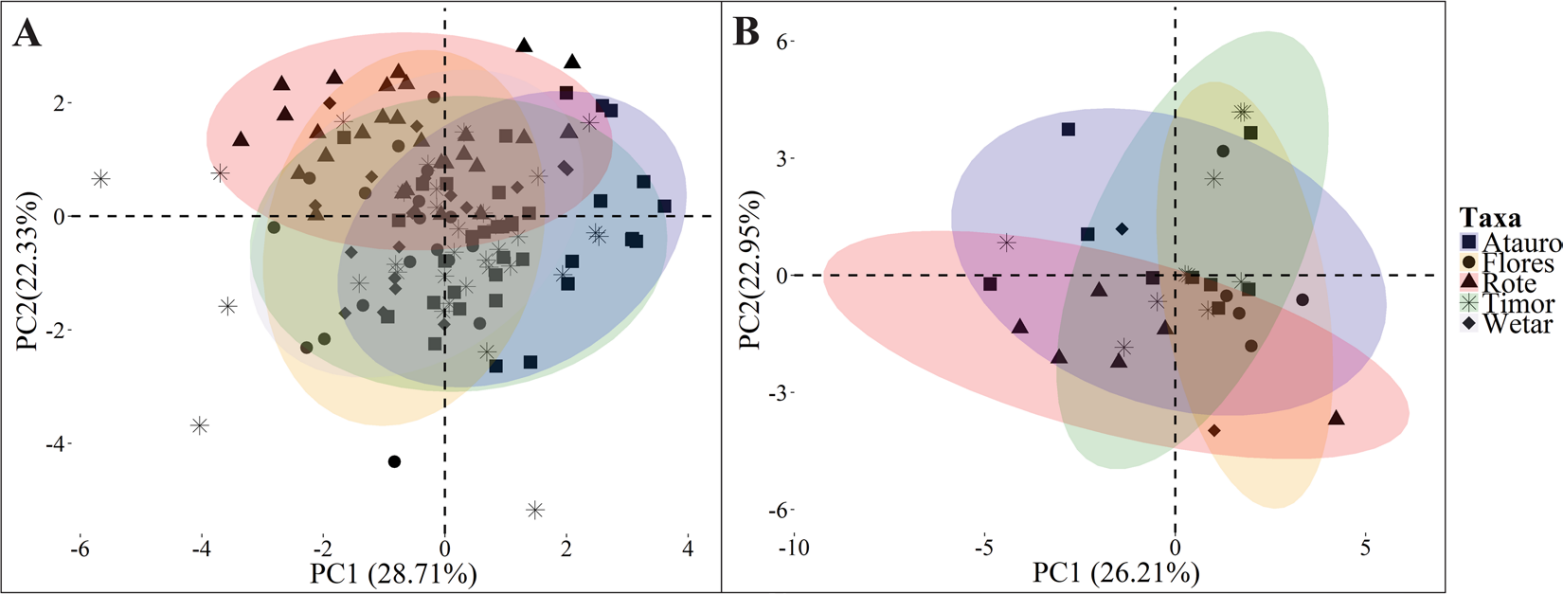
Principal component analysis plots of (**A**) songs [9 parameters] and (**B**) calls [19 parameters] of the Lesser Sundaic *Phylloscopus* leaf warblers. Ellipses represent 95% confidence intervals of each taxon.

PCA of 1,095 song and call strophes from across several populations of Lesser Sundaic *Phylloscopus* leaf warblers did not produce distinct bioacoustic clusters. Both vocalization types, calls and songs, exhibited extensive overlap among island populations in the first two PCs, which together accounted for ~50% of total observed variance (Fig. 7).

## Discussion

### Phenotypic and Genomic Distinctiveness

We present strong evidence that the Rote Leaf Warbler is morphologically and biogeographically highly distinct from all other leaf warblers across Wallacea and beyond. Morphologically, the Rote Leaf Warbler differs from all congeners – to the best of our knowledge – in its substantially longer bill (as scaled to body length), which in the field is reminiscent of the tailorbirds (*Orthotomus*), a trait that is unknown from any other leaf warbler (Fig. 3). Beyond bill shape, it can be distinguished from other Indo-Papuan leaf warblers based on a range of differences in plumage colouration, and from most by the colour and pattern of the lower mandible. We have also used population genomic and phylogenomic analyses in combination with thousands of loci from throughout the genome to show that the Rote Leaf Warbler is genomically distinct from its presumably most closely related congener, the geographically adjacent Timor Leaf Warbler (Figs. 4 and 6). A threshold of ~2% divergence in cytochrome C oxidase unit 1 (COI) is generally used to indicate species-level divergence in birds^44^. As COI in birds mutates at about 1.8% Ma^−1^, slower than the ~2.1% Ma^−1^ observed in cyt-b^42,43,45^, the species threshold in cytb thus translates to ~2.33%, which is substantially lower than the cytb divergences of 3.6% between the Rote and Timor Leaf Warblers. We show through application of the cytb mitochondrial clock that these two sister taxa likely diverged more than 1.7 million years ago.

Despite deep genomic and morphological differentiation setting apart the Rote Leaf Warbler from adjacent species, our bioacoustic analyses did not reveal vocal differentiation between different island taxa (Fig. 7). In extent and rigour, our vocal analysis is probably among the most comprehensive bioacoustic comparisons ever performed for Wallacean birds, comprising measurements of 19 parameters across 153 recordings, totalling 1,095 strophes and 7,446 elements. The massive breadth of our vocal inquiry notwithstanding, leaf warbler vocalizations are extremely variable and individually complex, and defy the most rigorous of attempts to find a homologous signal among the multitude of different vocal strophes^46^. Perhaps future studies utilising an even wider range of parameters may yet be able to better characterize the complex vocalizations of these variable songsters. Meanwhile, we have no doubt that the Rote Leaf Warbler warrants species-level recognition on account of its extraordinary morphological and genomic features.

### Behaviour and Ecology

Little is currently known about the behaviour and ecology of this species. Our observations, corroborated by reports from birdwatchers and researchers who have witnessed this species in the field (e.g., J.A. Eaton, pers. comm.) indicate that the Rote Leaf Warbler, like its congeners elsewhere, is highly active and is constantly in motion.

Regardless of habitat type the leaf warbler is observed in, it always inhabits the tallest available stratum of vegetation. In lower forest on the Tapuafu Peninsula (Fig. 1), the bird foraged in low to mid-sized trees between five and eight meters in height, even coming as low as breast height in areas devoid of taller trees. However, in the Seda forest patch (Fig. 1), which is composed of taller trees, it was observed to keep to the canopy, between 12 to 20 meters off the ground.

While many leaf warblers elsewhere are generally known to be leaf and foliage gleaners^1,8^, we observed the Rote Leaf Warbler to also be a bark gleaner, with insect prey actively taken from tree branches and trunks. Bark-gleaning behaviour could be the reason behind the unusually long bill of this taxon: a longer bill could confer evolutionary benefits by permitting the bird to more easily extract prey from between fissures in tree bark. It is difficult to predict why Rote Leaf-Warblers, but not any of the other island-inhabiting leaf-warblers from Wallacea, have evolved such an unusually long bill as a potential mechanism to widen their ecological niche. However, the great aridity of Rote Island, and its limited elevational spectrum as compared to virtually all other islands inhabited by leaf warblers in Wallacea, may be the primary reason. While other Wallacean islands are either more humid than Rote or provide higher elevations with rain-capturing topographic relief, Rote is more exposed to arid conditions, possibly making it more imperative for its native leaf warblers to adopt wider life-history strategies that go beyond the leaf-gleaning behavior typical for the genus. The bark-gleaning behavior of the leaf warblers on Rote, especially in the absence of any other avian bark-gleaners, may facilitate the long-term survival of these birds in an arid environment in which a sole reliance on leaf-gleaning may expose a species to higher extinction risk.

### Conservation

The Rote Leaf Warbler is endemic to the island of Rote in the province of East Nusa Tenggara, Indonesia, where it inhabits intact primary deciduous forest as well as secondary forest. Due to Rote Island’s long history of intensive agricultural use, there is little forest habitat on the island with the exception of the Tapuafu Peninsula and the Seda forest areas.

We propose that the Rote Leaf Warbler be given an IUCN categorization of “Vulnerable” under criteria B1 and B2 of the 2001 IUCN red list criteria (v3.1; available at www.iucnredlist.org). Only approximately 19% of Rote Island is estimated to be covered by forest habitat suitable for this leaf warbler^9^, with suitable habitat patches heavily fragmented by human development, arid low bush, and exposed land. Rote has a total land area of 1284 km^2^, which places the Rote Leaf Warbler’s maximum extent of occurrence and area of occupancy at 1284 km^2^ and 244 km^2^ respectively, with the Tapuafu Peninsula hosting the most intact fragments. These numbers suggest that the leaf warbler may possibly fall under the “Endangered” category; however we lack data on population size and trends for confirmation. We do note that the leaf warbler does not seem to exist at particularly high population densities and we do not expect the global population size to be very high. Additionally, Rote-Ndao regency (which consists primarily of Rote Island) experiences very high rates of human population growth, with an increase of 28% in the six years between 2010 and 2016^47^. These human population growth trends are expected to continue, bringing with them increased rates of road-building and land cover conversion and further decreasing the amount of habitat available to the Rote Leaf Warbler.

Avian endemism on Rote Island has long been underestimated, and the island hosts many species-level endemics^8,13,48,49^ as well as an additional number of threatened, range-restricted species (e.g. Yellow-crested Cockatoo *Cacatua sulphurea*, Timor Green Pigeon *Treron psittaceus*, Jonquil Parrot *Aprosmictus jonquillaceus*)^13^. Despite Rote’s northern peninsula (Rote Utara) being identified as a Key Biodiversity Area by assessments of Wallacean biodiversity, the area was not selected as a priority site for investment by the Critical Ecosystems Partnership Fund^13^. Rote does not presently have any major terrestrial protected area^50^; the creation of new protected area(s) covering the most intact forest patches in North Rote and Seda is recommended to secure the long-term survival of the Rote Leaf Warbler as well the many other endemic and threatened taxa Rote Island hosts.

## Electronic supplementary material

Supplementary Dataset 1

Supplementary Dataset 2

## Data Availability

The datasets generated during and/or analysed during the current study are available in the Sequence Read Archive repository, under BioProject accession number PRJNA450732.
